# Metabolic Syndrome and Associated Factors in Farmers in Southeastern Brazil: A Cross-Sectional Study

**DOI:** 10.3390/ijerph20146328

**Published:** 2023-07-08

**Authors:** Ana Clara Petersen Cremonini, Júlia Rabelo Santos Ferreira, Cleodice Alves Martins, Camila Bruneli do Prado, Glenda Blaser Petarli, Monica Cattafesta, Luciane Bresciani Salaroli

**Affiliations:** 1 Health Sciences Center, Federal University of Espírito Santo, Vitoria 29043900, Brazil; ana.cremonini@edu.ufes.br; 2 Graduate Program Collective Health, Health Sciences Center, Federal University of Espírito Santo, Vitoria 29043900, Brazil; julia.ferreira@edu.ufes.br (J.R.S.F.); cleodice.martins@edu.ufes.br (C.A.M.); prado.camilab@gmail.com (C.B.d.P.); glenda.petarli@ebserh.gov.br (G.B.P.); monica.cattafesta@ufes.br (M.C.); 3 Graduate Program Nutrition and Health, Health Sciences Center, Federal University of Espírito Santo, Vitoria 29043900, Brazil

**Keywords:** metabolic syndrome, farmers, rural population

## Abstract

(1) Background: Metabolic syndrome is a strong predictor of cardiovascular disease thus the objective of the study was to verify the prevalence of metabolic syndrome in farmers, as well as to verify the association with sociodemographic, work and lifestyle factors. (2) Methods: Cross-sectional, observational study, conducted with 790 individuals. For the diagnosis of metabolic syndrome, the National Cholesterol Education Program’s Adult Treatment Panel III (NCEP-ATP III) and International Diabetes Federation (IDF) criteria were used. Pearson’s chi-square test and binary logistic regression were used to verify factors associated with metabolic syndrome (3) Results: The prevalence of MS according to the IDF criteria was 16.3% overall, with 9.7% of women (95% CI: 6.66–16.16) and 6.6% of men (95% CI: 5.17–11.97). According to the NCEP/ATP III criterion, it was 12.3% overall, with 7.5% corresponding to women (95% CI: 6.62–13.13) and 4.8% to men (95% CI: 3.5–8.70). With regard to the conditions that make up metabolic syndrome, it was found that high density lipoprotein, high blood pressure and high waist circumference were the most prevalent. (4) Conclusions: The prevalence of metabolic syndrome is considerable in the population when compared to other regions, both rural and urban, in Brazil.

## 1. Introduction

Metabolic Syndrome (MS) is a set of risk conditions that manifest simultaneously in the same individual and are characterized by obesity, particularly in the central region of the body, systemic arterial hypertension (SAH), insulin resistance and dyslipidemia [1]. The development of this syndrome is complex and not fully understood; however, studies indicate that insulin resistance is the basis of the pathophysiology since it triggers changes in fatty acid metabolism resulting in a cascade of actions that are involved in chronic low-grade inflammation and oxidative stress [2].

According to data from the National Health Survey (PNS), the prevalence of MS among Brazilians is 38.4%, being higher in women, individuals with low education and the elderly [3]. This overall prevalence is similar to the results of the National Health and Nutrition Examination Survey (NHANES) 2011–2016, in which MS affected 34.7% of the US population [4]. Research conducted in the city of Vitória, capital of the state where the municipality of the present study is located, found that 29.8% of individuals had MS according to the National Cholesterol Education Program’s Adult Treatment Panel III NCEP/ATP III criteria, with no difference in sex, but with an increase to 48.3% in the age group from 55–64 years-old [5].

Regarding rural populations, a survey conducted in Cavunge, Bahia, found a scenario very close to the national one where 30% of the sample had MS; the frequency was higher in women (38.4%) compared to men (18.6%) and was higher in individuals aged over 45 years (41.4%) [6]. In another study, conducted in the Jequitinhonha Valley, Minas Gerais, 14.9% of the participants had MS, with female sex, obesity and age being factors that were independently associated with the syndrome [7]. In a systematic review with meta-analysis involving Brazilian adults in the last 10 years, a prevalence of metabolic syndrome of 34% in urban areas and 15% in rural areas was found. Being a high prevalence when compared to other countries [8]. Regarding Brazilian adolescents, it was concluded that the prevalence was low and similar for both sexes, being less than 3% [9].

It is worth mentioning that rural communities present risks inherent to work in the field, such as exposure to dangerous equipment concomitant to the non-use of personal protective equipment (PPE), toxic substances, excessive exposure to the sun and venomous animals [10,11]. All of these factors are aggravated by the difficulty of accessing a health diagnosis and treatment services in rural areas; a reduction in the possibility of early prevention and treatment is noted, as well as an increase in the chances of developing chronic diseases, a scenario that suggests worse health conditions and higher levels of disease among rural populations compared to other populations [12,13].

The context of the metabolic syndrome has been widely studied internationally and nationally, but recent research on its prevalence in the rural population is lacking, as this is a very difficult group to study due to its geographical conditions, and it is necessary to have knowledge about the prevalence of MS in this population as well. In this context, this study aimed to estimate the prevalence of MS in farmers in the southeastern region of Brazil, as well as to verify the association between sociodemographic factors, work and lifestyle.

## 2. Materials and Methods

### 2.1. Population and Study Design

This is an observational, analytical study with a cross-sectional design derived from a larger project funded by the Research Program of the Unified Health System (PPSUS), through the public notice FAPES/CNPq/Decit-SCTIE-MS/SESA nº 05/2015–PPSUS, entitled “Health conditions and associated factors: a study of farmers in Espírito Santo”, which was approved by the Research Ethics Committee of the Health Sciences Center of the Federal University of Espírito Santo, under number 1,856,331.

The target population of the study consisted of family farmers registered in the Family Health Program, of both sexes, whose main source of income was a family-based agriculture developed in the municipality of Santa Maria de Jetibá, Espírito Santo- Brazil that would perform full working activities for at least six months and were able to respond to the questionnaire used in the research. All participants signed the Free and Informed Consent Form.

The selection of crops was made through a stratified draw, considering the number of families per community health agent (CHA) and respecting the proportionality between regions. The inclusion criteria were as follows: being aged between 18 and 59 years old, not being pregnant, being a farmer as the main source of income and being in full-time employment for at least six months. In order to avoid the interdependence of information, only one individual per family was considered for the draw. For each CHA, a reserve amount was drawn in case of refusal of the first draw; the sample size calculation was performed using the Epidat program version 3.0 and a prevalence of 20% (5), sampling error of 2.5% and significance level of 95% were considered, totaling 790 cultivations.

### 2.2. Data Collection

Data collection took place from December 2016 to April 2017 on the premises of the health units in the municipality, by a team composed of five trained researchers, of which four were responsible for applying the test and hemodynamic evaluation, and one was responsible for clinical and anthropometric examinations. Blood collection took place right after the TCLE was signed by the study participant, followed by the application of the test and hemodynamic evaluation. In the end, the farmer underwent an anthropometric assessment, and was then released.

Waist circumference (WC) was measured with an inelastic Sanny model TR-4010^®^ (Sanny, São Paulo, Brazil) measuring tape. Participants were positioned standing, with arms extended along the body, the tape was positioned between the upper edge of the iliac crest and the last rib. Three non-consecutive measurements were taken, the first discarded and the average of the last two considered in the final measurement.

Approximately 10 mL of blood was collected, obtained through venipuncture after 12 h of fasting. The collected blood was divided into two tubes, one containing EDTA anticoagulant and the other without anticoagulant. The biological material was transported in a thermal container to the Clinical and Histopathological Analysis Laboratory, where the analyses were performed. For High density lipoprotein cholesterol (HDL-c), the method used was the enzymatic colorimetric method with the HDL-c Treatment Kit^®^ (InVitro Diagnóstica Ltda, Itabira, Brazil). Triglycerides were determined by the colorimetric enzymatic method with the Liquicolor mono^®^ Triglycerides Kit (InVitro Diagnóstica Ltda). Blood glucose was determined by the colorimetric enzymatic method with the Glucose Enzimática^®^ Kit (InVitro Diagnóstica Ltda).

The hemodynamic data collected were systolic blood pressure and diastolic blood pressure. The hemodynamic measurement followed the procedures used in the protocol of the Brazilian Guidelines on Hypertension [14]. Farmers were instructed not to consume food, alcoholic beverages and coffee thirty minutes before BP measurement; not to smoke thirty minutes before data collection, not to practice strenuous physical exercise in the previous sixty minutes and to empty the bladder before performing the test. Participants were seated, legs uncrossed and feet flat on the floor, back resting on the chair and relaxed. The right arm was positioned at heart level, supported, with the palm facing upwards, without the clothes forming a tourniquet around the arm.

Measurements were taken during the interview, with four repetitions per individual. The first measurement was discarded, and the fourth measurement was only used if the difference between the second and third measurements was greater than 4 mmHg. For the hemodynamic protocol, the pressure monitor Omron Automatic HEM-7200^®^ (Omron, São Paulo, Brazil) was used, duly calibrated and validated by INMETRO (National Institute of Metrology, Quality and Technology).

The sociodemographic, laboratory and habits data of and life were obtained through the application of a semi-structured questionnaire. The variables used in this study are gender, age, marital status, education, race/color and socioeconomic class. Occupational data included weekly working hours and land tenure. Lifestyle-related variables were body mass index (BMI), physical activity, smoking and alcohol consumption. All variables were collected by trained researchers, according to appropriate protocols. More details about the methodology applied in the research can be found in a previous study [13].

### 2.3. Diagnosis of MS

For the diagnosis of MS, the criteria of the International Diabetes Federation (IDF) and National Cholesterol Education Program’s Adult Treatment Panel III (NCEP) were used. In the first panel, the individual with abdominal obesity, obligatorily assessed by WC ≥ 84 cm for women or ≥94 for men, plus two of the following criteria: fasting blood glucose ≥ 100 mg/dL; SBP ≥ 130 mmHg or DBP ≥ 85 mmHg; TG ≥ 150 mg/dl and HDL-c < 40 mg/dL for men and <50 mg/dL for women [14]. The NCEP considers MS in the presence of at least three of the following criteria: WC > 102 cm for men or >88 cm for women; HDL-c < 40 mg/dL for men and <50 mg/dL for women, TG ≥ 150 mg/dL; SBP ≥ 130 mmHg and DBP ≥ 85 mmHg and fasting blood glucose ≥ 100 mg/dL. The use of antihypertensive, hypoglycemic and/or hypolipidemic drugs are considered in both criteria for MS, as they classify the individuals as having hypertension, diabetes and/or dyslipidemia, respectively [15].

### 2.4. Statistical Analysis

The descriptive analysis of the sample and presence of the syndrome was performed using frequency. To describe the study variables, absolute and relative frequencies were used. First, the difference in proportion between the variables was analyzed using Pearson’s chi-square test, and then the variables that showed statistical significance were adjusted in the logistic regression model to test whether there was an association with the outcome. Finally, to analyze the components of the syndrome, Pearson’s chi-square test was used to verify differences in proportion; the results are shown in a graph for better understanding.

Pearson’s chi-square test (X2) was performed to analyze differences in proportions between qualitative variables and syndrome components, with *p* < 0.05 being considered significant differences. Binary logistic regression was used to test associations between the independent variables and the outcome, including the variables that presented *p*-value ≤ 0.05 in the bivariate analysis in the model. For all of them, the assumptions of the absence of multicollinearity were tested (tolerance >0.1 and variance inflation factor <10); the minimum sample size for the number of variables in the model (>20 individuals per variable in the model and >5 cases in each category of variables) and absence of outliers. For the binary logistic regression analysis, the enter method was used, adopting the model with the highest fit according to the Hosmer–Lemeshow test (*p* > 0.05, closest to 1.0) and the 95% confidence interval.

All analyses were performed using the statistical program IBM SPSS Statistics 23 (Armonk, NY, USA: IBM Corp), adopting a significance level of α < 5%.

## 3. Results

### 3.1. Characteristics of the Study Population

Data from this study refer to 790 farmers, of which 413 (52.3%) are men and 377 (47.7%) are women. The largest proportion of individuals is in the age group from 31–40 years old (N = 231; 29.2%), lives with a partner (N = 678; 85.8%), has a low level of education (N = 533; 67.5%), declare themselves white (N = 702; 88.9%), are in socioeconomic class C (N = 395; 50.0%) and are overweight/obese (N = 430; 51%). The prevalence of MS was higher according to the IDF criteria and was prevalent in 9.7% of women (95% CI: 6.66–16.16) and 6.6% of men (95% CI: 5.17–11.97). According to the NCEP/ATP III criteria, the prevalence of MS was 7.5% in women (95% CI: 6.62–13.13) and 4.8% in men (95% CI: 3.5–8.70) (Table 1).

The prevalence of MS was higher according to the IDF criteria and was prevalent in 16.3% (95% CI: 13.9–19.0) of the population; among those who had the syndrome, 9.7% were women (95% CI: 6.66–16.16) and 6.6% were men (95% CI: 5.17–11.97). According to the NCEP/ATP III criteria, the overall prevalence of MS was 12.3% and in those with the syndrome, 7.5% correspond to women (95% CI: 6.62–13.13) and 4.8% correspond to men (95% CI: 3.5–8.70) (Table 1).

In Table 2, it is possible to observe that the prevalence of MS in individuals presents significant proportional differences between females and males (*p* = 0.006 NCEP and *p* = 0.003 IDF) and age (*p* < 0.001 NCEP and *p* < 0.001 IDF).

Table 3 shows a statistical difference in the variable “land ownership” according to the NCEP criteria (*p* = 0.045). Being underweight/eutrophic or overweight/obese presents a significant difference between the proportions in relation to the presence and absence of MS (*p* ≤ 0.001 NCEP and *p* ≤ 0.001 IDF). According to the IDF criterion, being an alcoholic or not presents a significant proportion of differences in relation to having MS or not (*p* = 0.024 IDF). Land ownership showed a significant difference according to the NCEP criteria (*p* = 0.045). BMI showed statistical differences in both criteria (*p* < 0.001). Finally, statistical differences were verified in the variable alcohol consumption, but only in the IDF criterion (*p* = 0.024) (Table 3).

### 3.2. Binary Logistic Regression between MS and Analyzed Variables

Table 4 shows the binary logistic regression analysis for MS according to NCEP. Being a woman increased the chances of having MS by 1.72 times when compared to men (OR 1.72; 95% CI 1.09–2.71; *p* = 0.019). Being in the age group from 41 to 50 years increases the chances of having MS by 3.41 times (OR 3.41; 95% CI 1.59–7.31; *p* = 0.002) and increases to 4.52 times (OR 4.52; 95% CI 2.09–9.75; *p* < 0.001) in those aged over 50 years. Being overweight or obese increases the chances of having MS by 3.71 times when compared to those with low weight or eutrophy (OR 3.71; 95% CI 2.17–6.34; *p* ≤ 0.001). Not being a landowner increases the chances of having MS by 1.70 times when compared to those who own land (OR 1.70; 95% CI 1.03–2. 80; *p* = 0.035).

Table 5 shows the binary logistic regression between the statistically significant variables according to the chi-square and MS (IDF). Women were 1.64 times more likely to have MS when compared to men (OR 1.64; 95% CI 1.06–2.55; *p* = 0.026). Individuals aged 41 to 50 years are 2.55 times more likely (OR 2.55; 95% CI 1.34–4.84; *p* = 0.004) to develop MS, increasing to 3.12 times (OR 3.12; 95% CI 1.62–6.02; *p* < 0.001) for those over 50 years old. Overweight or obese individuals are 5.01 times more likely to have MS when compared to those with low weight or eutrophy (OR 5.01; 95% CI 3.06–8.20; *p* < 0.001).

## 4. Discussion

Our findings show that the main risk factors related to MS in farmers are gender, age, BMI and land ownership and that the main attributes for the high prevalence of MS are obesity, the substitution of in natura foods for ultra-processed foods and the inflammatory profile. The findings of this study corroborate the results of the literature by noting that women, the elderly and the obese are more likely to have MS [3,6,7,9,16]. We also considered laboratory data for a more robust analysis of this condition and found that HDL, blood pressure and WC are the most prevalent components of MS, with a higher BP in men and high WC and lower HDL in women. These findings are valuable because they show the risk profile of farmers and break with the belief that “farmers enjoy better health”, by demonstrating the considerable prevalence of MS, classified as a strong predictor of cardiovascular diseases [17].

It is important to emphasize that work in the field is often related to an occupation model that promotes health and is often associated with the image of a fully healthy lifestyle, as it provides frequent exposure to nature, outdoors, physical exertion, greater proximity to in natura and nutritionally rich foods. However, our results demonstrate a different reality by noting a high prevalence of being overweight and obese, especially in the abdominal region, as well as hypertension and dyslipidemia, which constitute substantial risk factors for MS. This paradox has strong roots in the cultural transition observed between farmers in recent decades, especially in relation to food, since, although farmers are close to planting and harvesting food, this does not imply its consumption [18].

Recent epidemiological studies demonstrate that the main attribute related to the high prevalence of SM is the changes in rural eating habits by showing a greater substitution of in natura foods for processed and ultra-processed foods, which are calorie-dense and potentially obesogenic [19,20,21]. In a survey that analyzed the same population as the present study, it was found that 19.7% of rural residents are obese and other studies have found similar results by finding a high prevalence of being overweight among farmers, a profile that causes chronic inflammation. This inflammatory profile triggers multimorbidity that affects 41.5% of farmers; about 77% of them have more than one chronic disease [13].

In addition to changes in dietary patterns, technological changes and the modernization of work activities have resulted in changes in the lifestyle of rural residents, as a result of which there is damage to health. Studies show that these changes have increased workers’ exposure to the main risk factors for MS, especially the increase in dyslipidemia, excess weight and increased WC [22,23,24]. In a study conducted in southeastern Brazil, damage to psychological health was also reported, with the identification of a high prevalence of depressive symptoms among rural workers (16.8%), which are associated with not owning land, pesticide poisoning and professional dissatisfaction [25].

In our study, we verified that the female gender is associated with MS [3], which is a finding that corroborates other studies conducted in Brazil and other countries; in India, MS can affect three out of five women living in rural areas [26]. This high prevalence in females can be explained by hormonal variations, since estrogen regulates several aspects of lipid metabolism and insulin resistance [27].

With advancing age, especially after menopause, it is common to see a decrease in estrogen levels and the functioning of its receptors, causing dyslipidemias such as low HDL-c, greater predisposition to MS and cardiovascular diseases [28,29]. In addition, a less healthy lifestyle with an inadequate diet and a sedentary lifestyle, in addition to household responsibilities that culminate in an increase in women’s working hours, may exert an influence on the risk of becoming ill [3].

It can also be observed that MS is more prevalent in older individuals, which can be explained by physiological issues, since individuals naturally become more susceptible to the main risk factors for MS with aging, such as resistance to insulin, chronic inflammation and dyslipidemias [29]. The results of a study conducted in the United States are similar to ours, as they indicate significant discrepancies in relation to age; the prevalence of MS increased considerably with age, reaching more than twice the number of elderly people in relation to those aged 20–39 years [4]. Similar data were found using data from the China Nutrition and Health Surveillance (2015–2017) with more than 130,000 people, which also found a much higher prevalence of MS in older individuals, 44.2% in those aged ≥75 years and 23.3% in those aged between 20 and 44 years [17].

Being overweight/obese is one of the main risk factors of MS. These results are in line with that which was published by the Global Burden of Disease Study, which highlights the role of excess weight in the incidence of chronic diseases globally and states that being overweight is strongly associated with a sedentary lifestyle and the adoption of diets that are poor in natural foods and rich in processed foods; this pattern has become popular all over the world, especially in Latin America, where high BMI represents the main risk factor for the development of chronic diseases [30]. Thus, MS assumes a prominent role in this scenario, as it is a syndrome that is strictly related to obesity, especially the one characterized by the accumulation of fat in the abdominal region, which contributes to increased insulin resistance and cardiovascular diseases [29].

Our study showed that not owning land is a risk factor for MS, which seems to be related to sociocultural/financial issues. This is because Brazil is a country marked by land ownership concentration; with that, land ownership has been the object of constant claims by social movements that seek the right to land as a means of producing food for subsistence. In addition to food insecurity, according to a study conducted with members of the Movimento Sem Terra (MST), individuals who do not own land have precarious health conditions that are reflected by the high prevalence of infectious diseases, being overweight/obese and an increase in non-communicable, transmissible chronic diseases [31]. Added to this are geographic and socioeconomic factors that act as barriers because they make it difficult to access health services in distant regions. A study conducted in a remote rural community in India showed that the average time to access outpatient care and immunizations is about 1 h, increasing to almost 3 h for delivery and hospitalization points when walking. These values decrease to less than one hour and 1.5 h, respectively, when in a private four-wheel vehicle, thus revealing a factor of financial inequality [32].

Regarding the components of the syndrome, men and women have significant differences in proportion regarding HDL-c, BP and WC. Among the five variables used in the diagnosis, the difference in the proportion of WC for both diagnostic criteria stands out: it is noted that the prevalence of elevated WC is markedly higher in women, reaching 3x more women by the NCEP criterion and twice as many by the NCEP criteria IDF. This finding is alarming because although it is common for MS to affect more women than men, the difference between the sexes is very large, thus making it a considerable risk factor [3,17].

It is important to emphasize that our findings, despite being relevant results, are a study of a rural population from only one region of the Southeast. However, it is important to emphasize that our findings are in line with the results indicated by both a representative study of the Brazilian population that uses data from the National Health Survey and a systematic review of the prevalence of MS in Latin American countries. According to Oliveira et al., high WC and low HDL-c were the most prevalent components in Brazilians. Elevated WC was prevalent in more than half of the individuals, being considerably higher in women, and low HDL-c was prevalent in half of the individuals. In the systematic review covering countries such as Colombia, Peru, Mexico, Brazil, Venezuela and others, the most frequent components of MS were low HDL-c (62.9%) and abdominal obesity (45.8%) [33]. In addition to these studies conducted in America, surveys conducted in other regions also show similar results [9,17,34].

## 5. Conclusions

The prevalence of MS was considerable in this population when compared to other studies published in the literature. Thus, it is configured as an alert, requiring the maintenance and strengthening of measures aimed at reducing MS. Thus, it is essential to encourage healthy eating and reduce the consumption of industrialized foods, in addition to encouraging the practice of physical activity; these are the potentially most effective modifiable risk factors in preventing the components of MS and possibly reducing prevalence in the population. In addition, the results of this study can serve as a source of information about the health status of rural residents of the Brazilian population, especially when we consider the scarcity of studies on rural MS in Brazil. Thus, this theoretical framework can serve as a guide for the development of programs and specific public policies to combat MS in rural populations in Brazil as well as in other countries.

## Figures and Tables

**Table 1:** Sociodemographic characterization and BMI of the sample according to sex and prevalence of MS according to the NCEP and IDF criteria.

Variables	Male N (%) 95% CI	Female N (%) 95% CI	Total N (%) 95% CI
Age (years)			
<30	106 (13.4) 11.15–16.04	107 (13.5) 11.27–16.17	213 (26.9) 23.9–30.1
31–40	122 (15.4) 13.03–18.19	109 (13.8) 11.50–16.44	231 (29.2) 26.1–32.5
41–50	100 (12.7) 10.46–15.22	95 (12.0) 9.88–14.54	195 (24.7) 21.8–27.8
>50	85 (10.8) 8.72–13.18	66 (8.4) 6.56–10.56	151 (19.2) 16.5–22.0
Marital Status			
Single	48 (6.1) 4.55–8.03	11 (1.4) 0.73–2.55	59 (7.5) 5.8–9.4
Lives maritally	345 (43.7) 40.18–47.21	333 (42.1) 38.69–45.68	678 (85.8) 83.9–88.1
Does not live maritally	20 (2.5) 1.59–3.95	33 (4.2) 2.93–5.88	53 (6.7) 5.1–8.6
Education (Years)			
<4	273 (34.5) 31.26–38.00	260 (33.0) 29.66–36.32	533 (67.5) 64.1–70.7
4–8	96 (12.1) 9.99–14.68	77 (9.7) 7.81–12.08	173 (21.9) 19.1–24.9
>8	44 (5.6) 4.12–7.46	40 (5.1) 3.68–6.89	84 (10.6) 8.6–12.9
Race/color			
White	362 (45.9) 42.31–49.37	340 (43.0) 39.56–46.57	702 (88.9) 86.5–90.9
Not white	51 (6.5) 4.88–8.45	37 (4.6) 3.36–6.46	88 (11.1) 9.1–13.5
Socioeconomic Class			
A/B	42 (5.3) 3.90–7.17	16 (2.0) 1.20–3.34	58 (7.3) 5.7–9.3
C	222 (28.1) 25.01–31.40	173 (21.9) 19.09–24.98	395 (50) 45.5–53.5
D/E	149 (18.9) 16.22–21.80	188 (23.8) 20.89–26.95	337 (42.7) 39.2–46.1
BMI			
Low weight/eutrophy	217 (27.5) 24.40–30.74	170 (21.5) 18.73–24.58	387 (49.0) 45.5–52.5
Overweight/obesity	196 (25.0) 21.86–28.00	207 (26.0) 23.19–29.44	403 (51.0) 47.5–54.5
Presence of MS			
NCEP	38 (4.8) 3.5–8.70	59 (7.5) 6.62–13.13	97 (12.3) 10.1–14.7
IDF	52 (6.6) 5.17–11.97	77 (9.7) 6.66–16.16	129 (16.3) 13.9–19.0

BMI: body mass index; MS: metabolic syndrome; NCEP: National Cholesterol Education Program’s Adult Treatment Panel III and IDF: International Diabetes Federation.

**Table 2:** Prevalence of metabolic syndrome, according to NCEP and IDF criteria, and proportional differences between sociodemographic characteristics in farmers—southeastern Brazil.

Variables	NCEP Criteria		*p*-Value	IDF Criteria		*p*-Value	Total *n* (%)
	Absent	Present		Absent	Present		
	*n* (%)	*n* (%)		*n* (%)	*n* (%)		
Gender							
Male	375 (47.5)	38 (4.8)	**0.006**	361 (45.7)	52 (6.6)	**0.003**	413 (52.3)
Female	318 (40.3)	59 (7.5)		300 (38.0)	77 (9.7)		377 (47.7)
Age (years)							
≤30	203 (25.7)	10 (1.3)	**<0.001**	197 (24.9)	16 (2.0)	**<0.001**	213 (27.0)
31–40	209 (26.5)	22 (2.8)		199 (25.2)	32 (4.1)		231 (29.2)
41–50	163 (20.6)	32 (4.1)		154 (19.5)	41 (5.2)		195 (24.7)
≥50	118 (14.9)	33 (4.2)		111 (14.1)	40 (5.1)		151 (19.1)
Marital status							
Single	52 (6.6)	7 (0.9)	0.545	51 (6.5)	8 (1.0)	0.833	59 (7.5)
Lives maritally	592 (74.9)	86 (10.9)		566 (71.6)	112 (14.2)		678 (85.8)
Does not live maritally	49 (6.2)	4 (0.5)		44 (5.6)	9 (1.1)		53 (6.7)
Education (years)							
<4	460 (58.2)	73 (9.2)	0.148	436 (55.2)	97 (12.3)	0.055	533 (67.5)
4–8	159 (20.1)	14 (1.8)		155 (19.6)	18 (2.3)		173 (21.9)
>8	74 (9.4)	10 (1.3)		70 (8.9)	14 (1.8)		84 (10.6)
Race/color							
White	616 (78.0)	86 (10.9)	0.946	590 (74.7)	112 (14.2)	0.421	702 (88.9)
Not white	77 (9.7)	11 (1.4)		71 (9.0)	17 (2.2)		88 (11.1)
Socioeconomic class							
A/B	54 (6.8)	4 (0.5)	0.297	52 (6.6)	6 (0.8)	0.424	58 (7.3)
C	341 (43.2)	54 (6.8)		330 (41.8)	65 (8.2)		395 (50.0)
D/E	298 (37.7)	39 (4.9)		279 (35.4)	58 (7.3)		337 (42.7)

MS: Metabolic Syndrome; NCEP: National Cholesterol Education Program’s Adult Treatment Panel III; IDF: International Diabetes Federation. Chi-square test. In bold: statistically significant values (*p* < 0.05).

**Table 3:** Prevalence of metabolic syndrome, according to NCEP and IDF criteria, and proportional differences between work and lifestyle characteristics in farmers—southeastern Brazil.

Variables	NCEP Criteria		*p*-Value	IDF Criteria		*p*-Value	Total *n* (%)
	Absent	Present		Absent	Present		
	*n* (%)	*n* (%)		*n* (%)	*n* (%)		
Hours of work per week							
<40	136 (17.2)	26 (3.3)	0.101	128 (16.2)	34 (4.3)	0.072	162 (20.5)
>40	557 (70.5)	71 (9.0)		533 (67.5)	95 (12.0)		628 (79.5)
Land tenure							
Owner	542 (68.6)	67 (8.5)	**0.045**	516 (65.3)	93 (11.8)	0.140	609 (77.1)
Non-owner	151 (19.1)	30 (3.8)		145 (18.4)	36 (4.6)		181 (22.9)
BMI							
Low weight/eutrophy	368 (46.6)	19 (2.4)	**<0.001**	365 (46.2)	22 (2.8)	<0.001	387 (49.0)
Overweight/obesity	325 (41.1)	78 (9.9)		296 (37.5)	107 (13.5)		403 (51.0)
Physical activity (PA)							
Don’t practice PA	565 (71.5)	80 (10.1)	0.967	539 (68.2)	106 (13.4)	0.979	645 (81.6)
Practice PA (<150 min) *	107 (13.5)	14 (1.8)		102 (12.9)	19 (2.4)		121 (15.3)
Practice PA (>150 min) *	21 (2.7)	3 (0.4)		20 (2.5)	4 (0.5)		24 (3.0)
Smoking							
Non-smoking	585 (74.1)	80 (10.1)	0.845	558 (70.6)	107 (13.5)	0.550	665 (84.2)
Currently smoker	53 (6.7)	9 (1.1)		49 (6.2)	13 (1.6)		62 (7.8)
Smoker in the past	55 (7.0)	8 (1.0)		54 (6.8)	9 (1.1)		63(8.0)
Alcoholism							
No	382 (48.4)	61 (7.7)	0.149	359 (45.4)	84 (10.6)	0.024	443 (56.1)
Yes	311 (39.4)	36 (4.6)		302 (38.2)	45 (5.7)		347 (43.9)

MS: Metabolic syndrome; NCEP: National Cholesterol Education Program’s Adult Treatment Panel III; IDF: International Diabetes Federation; BMI: body mass index; PA: Physical activity. *: less or more than 150 min a week. Chi-square test. In bold: statistically significant values (*p* < 0.05).

**Table 4:** Logistic regression analysis, including crude and adjusted OR, for the syndrome based on the NCEP criteria.

Variables	Crude Model			Adjusted Model		
	*p*-Value	OR	95% CI	*p*-Value	OR	95% CI
Gender						
Male		1			1	
Female	**0.006**	1.183	1.18–2.82	**0.019**	1.722	1.09–2.71
Age (years)						
<30		1			1	
31–40	0.054	2.137	0.98–4.62	0.113	1.893	0.86–4.16
41–50	**<0.001**	3.985	1.90–8.34	**0.002**	3.418	1.59–7.31
>50	**<0.001**	5.677	2.70–11.93	**<0.001**	4.525	2.09–9.75
BMI						
Low weight/eutrophy		1			1	
Overweight/obesity	**<0.001**	4.648	2.75–7.84	**<0.001**	3.711	2.17–6.34
Land tenure						
Owner		1			1	
Non-owner	**0.046**	1.607	1.00–2.56	**0.035**	1.706	1.03–2.80

OR: odds ratio; CI: confidence interval; values in bold: significant results.

**Table 5:** Logistic regression analysis, including crude and adjusted OR, for the syndrome based on the IDF criteria.

Variables	Crude Model			Adjusted Model		
	*p*-Value	OR	95% CI	*p*-Value	OR	95% CI
Gender						
Male		1			1	
Female	**<0.003**	1.782	1.21–2.61	**0.026**	1.647	1.06–2.55
Age (years)						
<30		1			1	
31–40	0.034	1.980	1.05–3.72	0.129	1.657	0.86–3.18
41–50	**<0.001**	3.278	1.77–6.06	**0.004**	2.558	1.34–4.84
>50	**<0.001**	4.437	2.37–8.28	**<0.001**	3.127	1.62–6.02
BMI						
Low weight/eutrophy		1			1	
Overweight/obesity	**<0.001**	5.997	3.69–9.72	**<0.001**	5.014	3.06–8.20
Alcoholic						
No		1		1		
Yes	**0.024**	0.637	0.43–0.94	0.655	0.902	0.57–1.41

OR: odds ratio; CI: confidence interval; values in bold: significant results.

## Data Availability

The data presented in this study are available in article.
